# The interrelation between proactive coping and job stressors subjective evaluation in healthcare professionals during the early phase of the COVID-19 pandemic

**DOI:** 10.1192/j.eurpsy.2021.842

**Published:** 2021-08-13

**Authors:** A. Kuznetsova, M. Gushchin

**Affiliations:** Faculty Of Psychology, Lomonosov Moscow state university, Moscow, Russian Federation

**Keywords:** proactive coping, Chronic Stress, job stressors, COVID-19

## Abstract

**Introduction:**

Proactive coping helps to reduce stress “in advance” – by possible stressors’ anticipating (Greenglass & Fiksenbaum, 2009). Does it helps to reduce distress in hazardous work environment with extremely high uncertainty level – like in healthcare professionals’ work at the beginning COVID-19 pandemic? Data showed the lover level of proactive coping in healthcare professionals in comparison with non-medical group (Pearman, Hughes, Smith & Neupert, 2020). The acute issue is to investigate proactive coping among medical professionals with different stress level.

**Objectives:**

Specialists of Moscow public dispensaries (doctors, n=209; nurses, n=131) were checked during pandemic breakout (April 2020) - in order to compare proactive coping and job stressors’ subjective evaluation in groups with high and low chronic states.

**Methods:**

The diagnostic set included: the job stress survey (Spielberger, 1994); the proactive coping inventory (Greenglass, 2002); the chronic stress and fatigues inventories (Leonova, 2012).

**Results:**

Cluster analysis by combination of stress-fatigue scores extracted equal 22% of professionals in risk subgroups. Surprisingly no proactive coping differences were found in nurses; among doctors preventive coping is significantly lower in risk subgroup (t=7.05; p=0.009). Revealed job stressors in risk groups for nurses are quite typical; but for doctors they are unusual: extreme workload (t=33.97; p<0.001), low coworkers support (t=48.94; p<0.001), lack of positive feedback (t=62.29; p<0.001).

**Conclusions:**

Despite the undeniable workload increase, well-to-do professionals perceived no high job stressors. In risk subgroup with lack of preventive coping, perceived stressors are likely connected with inability to predict strain increase and to minimize the impact of its negative effects (Moore, 2017).

